# EVIDENCE-INFORMED ONLINE PLATFORMS FOR DEMENTIA CAREGIVERS

**DOI:** 10.1093/geroni/igae098.2036

**Published:** 2024-12-31

**Authors:** Alan Stevens, Sara Czaja

**Affiliations:** Chair; Discussant

## Abstract

Online platforms for dementia care have emerged in response to the expressed desire of family caregivers for on-demand, self-paced education and dementia care skills training. Given the wealth of evidence-based dementia caregiver interventions, there is a unique opportunity to provide evidence-informed online services for family caregivers. Four online platforms for dementia caregivers will be discussed. Dr. Stevens will present an overview of two online platforms, GamePlan4Care (GP4C) and Resoures4Care (R4C). Preliminary findings suggest positive benefits to caregivers who engaged in either platform. The GP4C online platform has been modified to address the needs of family caregivers who experience the hospitalization of a Veteran living with dementia by Dr. Horstman. Preliminary findings from Dr. Horstman’s pilot study of GP4-Hospital in a VA hospital will be presented. Dr. Laura Gitlin’s presentation will focus on WeCareAdvisor, an online platform that provides daily tips to caregivers. Dr. Gitlin will present findings on the impact of multiple techniques used to engage family caregivers in using WeCareAdvisor. Dr. Eric Jutkowitz will provide an overview of Plans4Care, a translation of therapeutic strategies that address caregiver challenges from multiple in-person evidence-based dementia caregiver interventions into a single digital format. His presentation will focus on innovative online methods of tailoring therapeutic content to family caregivers. Dr. Sara Czaja, an international leader in developing, implementing, and evaluating technology-based solutions to late-life autonomy and caregiving support, will frame the findings of the four presentations within the US landscape of online support services for dementia caregivers.

